# Tissue healing changes on wounds in rats after treatment with *Hancornia speciosa* latex in cream-gel formulation

**DOI:** 10.1590/acb371001

**Published:** 2022-12-19

**Authors:** Patrícia Lima D’Abadia, Susy Ricardo Lemes, Paulo Roberto de Melo-Reis, Ruy de Souza Lino, Pablo José Gonçalves, Diego dos Santos Reis, Graziele Alícia Batista Caixeta, Vanessa Cristine Santana Amaral, Luciane Madureira Almeida

**Affiliations:** 1 Graduate student. Universidade Estadual de Goiás – Programa Recursos Naturais do Cerrado – Biotechnology Laboratory – Anápolis (GO), Brazil.; 2 PhD, Assistant professor. Centro Universitário Goyazes – Department of Agricultural Science – Trindade (GO), Brazil.; 3 PhD, Assistant professor. Pontifícia Universidade Católica de Goiás – Biomedicine Department – Laboratory of Experimental and Biotechnological Studies – Goiânia (GO), Brazil; 4 PhD, Associate professor. Universidade Federal de Goiás – Experimental Pathology Laboratory – Institute of Tropical Pathology and Public Health – Goiânia (GO), Brazil.; 5 PhD, Associate professor. Universidade Federal de Goiás – Institute of Physics – Goiânia (GO), Brazil.; 6 Graduate student. Universidade Estadual de Goiás – Laboratory of Pharmacology and Toxicology of Natural and Synthetic Products – Anápolis (GO), Brazil.; 7 Graduate student. Universidade Estadual de Goiás – Sciences Applied to Health Products – Laboratory of Pharmacology and Toxicology of Natural and Synthetic Products – Anápolis (GO), Brazil.; 8 PhD, Full professor. Universidade Estadual de Goiás – Laboratory of Pharmacology and Toxicology of Natural and Synthetic Products – Anápolis (GO), Brazil.; 9 PhD, Full professor. Universidade Estadual de Goiás – Biotechnology Laboratory – Anápolis (GO), Brazil.

**Keywords:** Collagen, Wound Healing, Apocynaceae, Rats

## Abstract

**Purpose:**

*Hancornia speciosa* latex has shown pharmacological potential in wound healing processes due to its angiogenic, osteogenic, and anti-inflammatory activities. The aims of this study were to carry out a cream-gel formulation with 5, 10 and 25% of *H. speciosa* serum latex and to evaluate its potential to stimulate the skin regeneration in rats’ wounds.

**Methods:**

One hundred and twenty rats were divided into five groups: neutral control with saline (G1), cream-gel based on *H. speciosa* latex serum at 5% m/v (G2), cream-gel at 15% m/v (G3), cream-gel at 25% m/v (G4), and cream-gel (G5). The animals were euthanized at three, seven, 14 and 21 days after the injury induction, and some parameters were analyzed: wound contraction, necrosis, fibrin, polymorphonuclear and mononuclear infiltrates, fibroblast, angiogenesis, hemorrhage, and collagen.

**Results:**

The therapeutic treatment with cream-gel at 15 and 25% is beneficial in the inflammatory phase of healing processes since it increased the angiogenesis and proliferation of mononuclear infiltrations in wounds. Regarding wound contraction, the treatment with cream-gel (5 and 15%) induced a higher rate of contraction in the proliferative phase. The 15% cream-gel formulation stimulated a greater production of collagen in the injured tissues.

**Conclusions:**

*H. speciosa* cream-gel is a low-cost herbal medicine which can aid in tissue repair.

## Introduction

The skin is one of the most vital organs in the human body since it operates as a protective barrier against external agents, as well as a temperature regulator^1^. Skin wounds are any injuries in the normal structure of the skin^2^ that can vary from a simple, acute wound to a chronic wound. Chronic wounds affect millions of patients worldwide (about 1-2% of the population) making them a global medical concern with high costs for health systems^3^. Synthetic drugs and different chemical substances have been commonly used for the treatment and healing of skin wounds, but, in the case of chronic wounds, these substances usually have functional limitations and side effects^4^. Therefore, the use of more sophisticated technologies, such as nanoparticles^5^, growth factors^6^, extracellular matrix proteins^7^, skin grafts^8^, stem cells^9^, oxygen therapy^10^, and negative pressure therapy^11^ have become more and more frequent. These products and technologies have different indications and action mechanisms at different stages of the healing process. Despite their benefits, all the methods mentioned have high cost, and the effectiveness of these treatments is still debated within the scientific community^12^^,^ 13. Currently, there is no definitive and effective treatment for chronic skin wounds on the market.

Given this scenario, the development of new strategies using natural products and their bioactive components have been intensively studied as alternative sources of medicines for tissue regeneration^4^^,^ 14 ^-^ 16. The main advantage of using natural products is the low cost when compared to conventional products for the treatment of wounds. Based on previous ethnobotanical knowledge^17^, we decided to investigate the healing potential of *Hancornia speciosa* latex.

*Hancornia speciosa* Gomes (Apocynaceae) is a plant native to Brazil, found in a variety of landscapes such as coastal plains, the northeastern plains, the *cerrado* in the Midwest, and in areas of the North and Southeast^17^. Latex from this species, popularly known as *mangabeira*, is used as herbal medicine for treatment of dermatoses, tuberculosis, gastric ulcers, fractures, and fungal diseases^17^^-^ 19. Literature data report that this natural compound is biocompatible, non toxic^20^^,^ 21 and has marked angiogenic properties^22^^,^ 23, as well as osteogenic^24^, anti-inflammatory^25^, antioxidant,^23^ and antifungal^26^. These properties suggest that *H. speciosa* latex could be explored as source of new phytomedicines for wound treatment.

The aims of this study were to develop a cream-gel formulation with 5, 10 and 25% of *H. speciosa* serum latex and to evaluate its potential to stimulate the skin regeneration process in rats’ excisional wounds. Previous studies using *H. speciosa* latex biomembrane have shown the regenerative potential of this material^27^^,^ 28, but the cream-gel formulation has not been tested yet. The advantage of cream-gel formulation is the more efficient application property in comparison with latex biomembrane. For cream-gel preparation, we selected the *H. speciosa* serum fraction because previous studies associated this part with angiogenic activity.

## Methods

### Ethics concern

This study was conducted according to ethical standards after approval by the Ethics Committee on the Use of Animals of the Pontifícia Universidade Católica de Goiás (protocol 1630230718).

### Latex extraction

The latex of *H. speciosa* was collected from Universidade Estadual de Goiás tree collection, in the city of Ipameri, state of Goiás, Brazil. A voucher specimen was deposited at the Herbarium of Universidade Estadual de Goiás, Anápolis, Goiás, Brazil, under code number 4,875. The latex was collected in a sterile container by drilling into the tree trunk^29^.

### *Hancornia speciosa* latex fractionation

*Hancornia speciosa* latex was centrifuged at 4 °C for 1 h at 22,000 g (Heraeus Megafuge 16R, Thermo Scientific). After latex centrifugation, it was possible to obtain two different fractions:

- the top white zone with the rubber particles;
- the serum layer containing large variety of proteins and luteoid compounds.

The focus of this study was the serum fraction. After centrifugation, the rubber fraction was discarded.

### Formulation of Hancornia speciosa latex-based cream-gel

The *H. speciosa* latex serum was incorporated in a pharmaceutical base to produce a cream-gel which was used in experiments with excisional wounds on the backs of rats. The dry touch cream-gel composed of focus gel 305 (Infinity Pharma^®^) 5% self-emulsifying base, EDTA 0.2%, Nipagim 0.2% and distilled water was commercially obtained. The formulation was prepared by adding the aqueous phase (*H. speciosa* latex serum) to this commercial base, by manual shaking until complete homogenization was achieved. Three concentrations of latex serum in base (5, 15 and 25% m/v) were prepared. These concentrations were chosen because they did not significantly change the viscosity of the cream-gel and they are economically viable. The formulated cream-gel was stored in a refrigerator at 2–8 °C before application to the wound.

### Rats skin wounds model

One hundred and twenty male Wistar rats (*Rattus norvegicus albinus*) from 2 to 3 months old and body weight between 200 and 300 grams were used. The animals were acclimated in cages (three per cage), and the luminosity, temperature, noise intensity, and relative humidity of the air were those of the general environment. Water and commercial feed for the species were offered *ad libitum* to the animals.

### Experimental groups

For the accomplishment of this study, 120 male rats were randomly divided into five groups with six animals for each experimental exposition time (three, seven, 14, and 21 days after the injury induction – DAI):

- G1: animals treated with saline solution (0.9% saline solution);
- G2: cream-gel based on *H. speciosa* latex serum at 5% concentration;
- G3: cream-gel based on *H. speciosa* latex serum at concentration of 15%;
- G4: cream-gel based on *H. speciosa* latex serum at concentration of 25%;
- G5: cream-gel without latex.

For analysis of the proposed parameters, six animals from each experimental group were euthanized at three, seven, 14, and 21 DAI for morphometric and histological analysis of the injured area.

### Experimental procedure

The healing action of the cream-gel was evaluated in the model of excisional wound healing in rats, according to the methodology described by Garros *et al*.^30^, with some adaptations. Initially, the rats were anesthetized intramuscularly with ketamine (70 mg/kg, União Química Farmacêutica S/A, Brazil) and xylazine (10 mg/kg, Sespo Indústria e Comércio LTDA, Brazil) before surgical incisions. After injection of anesthetics, the animals were placed in a prone position, immobilized on a board, and subjected to trichotomy of the dorsal region with a blade. A mold was used to demarcate the wounds with an area equivalent to 4 cm^2^ (2 cm × 2 cm). Then, the excision of skin and subcutaneous tissue fragment from the demarcated area was performed with the aid of a scalpel and surgical scissors.

After the procedure, the animals were placed under observation for 1 hour, and analgesia was started with dipyrone 500 mg (30 mL/kg, Neo Química LTDA, Brazil), administered in the water provided *ad libitum* for seven days. The topical treatments of each group were applied immediately after the excisional wounds were made, in equal amounts and with enough to cover all the lesions once a day, during the 21 days of the experiment. In addition, sterilized occlusive dressings were used during the period in question (gauze and adapted dressing made with cotton wool) to keep the wound closed, providing ideal humidity, and making it difficult for other animals in the same cage to further injure the wound. The dressings were made daily and at the same time.

### Morphometric and histopathological evaluation

The healing process was evaluated through macro and microscopic observation of the lesions. In the end of each period, the animals in each group (zero, three, seven, 14, and 21 DAI) and the lesions were photographed for macroscopic evaluation. The photos of injuries allowed the analysis of presence of crust in a semi-quantitative way, according to the following criteria: absent, discreet (involvement of up to 25% of the area), moderate (from 26 to 50% of the area) and accentuated (over 50% of the area). The photographs were transferred to a computer and analyzed using the ImageJ 1.3.1 software. To determine the degree of contraction of the wound area, the formula proposed by Oliveira *et al*.^31^ was used (Eq. 1):


Relative wound contraction (%) = [(Initial injured area - contracted injured area) / Initial injured area] × 100% (1)

Subsequently, for each DAI (three, seven, 14, 21), six animals from each experimental group were euthanized with intraperitoneal thiopental (90 mg/kg) to perform a biopsy of the injured tissue and analysis of histopathological parameters. In this procedure, a sample was taken, which contained around 50% of the injured tissue and 50% of intact skin, with a safety margin of 1 cm around the center of the wound. The collected samples were fixed in 10% formaldehyde. The fragments of skin wounds fixed in 10% formaldehyde were processed according to routine histological technique, embedded in paraffin, from which slides stained with hematoxylin and eosin (HE) were made.

Histopathological analysis was performed blindly by a single observer under a brightfield microscope (Zeiss) at 40× magnification. The following parameters were observed: necrosis, fibrin, polymorphonuclear infiltrates (PMN), mononuclear infiltrates (MN), fibroblasts, angiogenesis, and hemorrhage. The scores were determined according to intensity:

- 0: absent;
- 1: mild (1-25% of the total area);
- 2: moderate (26-50% of the area);
- 3: stressed (51-100% of the area).

Slides stained with Picrosirius were also produced for the quantification of collagen fibers present in the samples^32^. For this purpose, images of the slides were captured using a polarized light microscope (Zeiss) coupled to a digital camera (Sony NEX-3). Images were recorded at 40× magnification from the beginning of the wound edge to the end of the other edge, being considered as valid fields those ones covering the area of the epidermis, superficial dermis, and deep dermis. The analysis of the photographs was performed using the ImageJ 1.3.1 software, which identified the collagen fibers in each field, through image binarization (Threshold/Cie-Lab function), and determined the percentage of the area.

### Statistical analysis

Statistical analysis of macroscopic data followed a normal distribution according to Shapiro-Wilk’s test of normality (R statistical program). Thereafter, the comparisons were performed using the one-way analysis of variance (ANOVA) test and the Tukey’s test. Results were considered significant when p < 0.05. Microscopic data, which do not follow a normal distribution, were evaluated using the non-parametric Kruskal-Wallis’ test, followed by multiple comparisons using the Dunn’s method, with the significance level of p < 0.05. Statistical analyses were performed using the R statistical program.

## Results

### Macroscopic analysis

**Figure 1:**
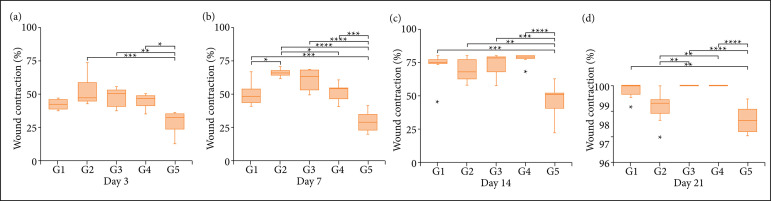
Wound contraction (percentage) of excisional wounds experimentally induced in rats and treated with *Hancornia speciosa* latex-based cream-gel. Statistical test: one-way analysis of variance (ANOVA) test and Tukey’s post-test, with significance level of p < 0.05.

DAI: days after the injury induction; M: mean; G1: neutral control (saline solution); G2: 5% cream-gel; G3: 15% cream-gel; G4: 25% cream-gel; G5: cream-gel.

**Figure 2:**
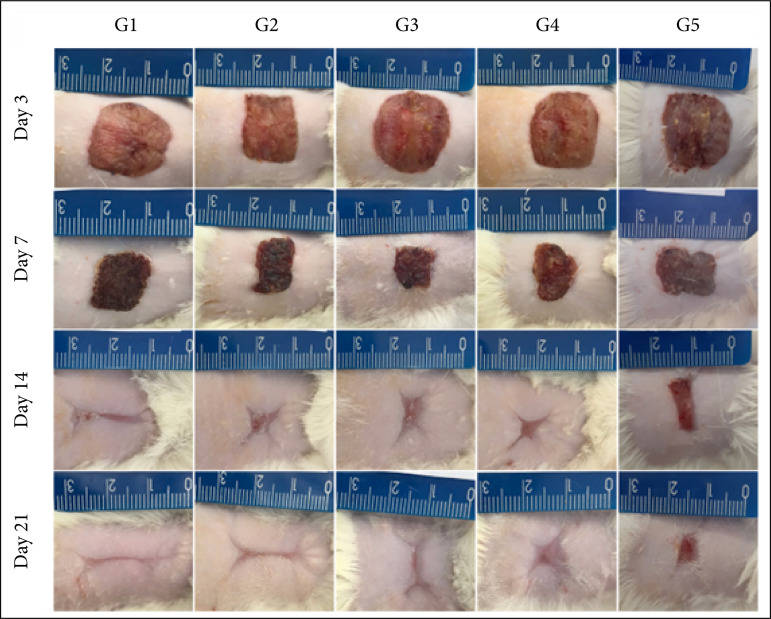
Macroscopic evolution of the excisional wounds experimentally induced in rats at three, seven, 14 and 21 days after the injury induction.

G1: neutral control (saline solution); G2: 5% cream-gel; G3: 15% cream-gel; G4: 25% cream-gel; G5: cream-gel.

Necrosis and re-epithelialization rates showed no statistical differences between G1, G2, G3 and G4 treatment groups (p > 0.05), however G5 showed significantly higher necrosis rate and lower re-epithelialization. Wound contraction rates were significantly higher in G3 and G4 than in G1 at seven DAI (p < 0.05), while no statistical difference was observed in three, 14 and 21 DAI (Fig. 1). Figure 2 shows representative images of evolution of excisional wounds in rats, in the groups treated with saline and cream-gels at concentrations of 0 (neutral control), 5, 15, and 25%.

### Microscopic analysis

Histopathological analysis of tissues removed from wounds performed in all treatment groups is shown in Table 1. In the inflammatory phase, at three DAI, statistical analyses showed that there was a significant difference between the groups. The group treated with cream-gel (G5) formed more fibrin than G1, G2 and G3. In addition, the group treated with 15% cream-gel (G3) formed more fibrin than the group treated with 5% cream-gel (G2). Despite the significant difference between G2 and G3 for fibrin formation, these groups did not differ from the control group (saline solution).

**Table 1:** General pathologic processes observed in excisional wounds experimentally induced in ratsand treated with *Hancornia speciosa* latex-based cream-gel*.

Pathologic processes DAI	G1	G2	G3	G4	G5	p
Necrosis 3	2	2	2.5	3	3	0.0312 G5 > G2
7	3	3	1.5	3	3	0.0691
14	0	0	0	0	1.5	0.0177 G5 > G2; G5 > G4
21	0	0	0	0	0	1.00
Fibrin 3	2.5	1	3	2	3	0.0138 G3 > G2
7	0	0	0.5	1	3	0.0026 G5 > G1; G5 > G2; G5 > G3
4	0	0	0	0	0	0.5915
21	0	0	0	0	0	1.00
Polymorphonuclear 3	2	2	2.5	3	3	0.0312 G5 > G2
7	3	3	1.5	3	3	0.0691
14	0	0	0	0.5	3	0.0022 G5 > G1; G5 > G2; G5 > G3; G5 > G4
21	0	0	0	0	2	0.0002 G5 > G1; G5 > G2; G5 > G3; G5 > G4
Mononuclear cells 3	1	1	2	2	2	0.0002 G3 > G1; G5 > G1; G3 > G2; G5 > G2
7	3	3	3	3	3	1.00
14	2	2	2	2	2	0.9908
21	1	1	1	1	1.5	0.1261
Fibroblast 3	1	1	2	2	1	0.0243 G3 > G1
7	3	3	3	3	3	1.00
14	3	3	3	3	2	< 0,0001 G1 > G5; G2 > G5 G3 > G5; G4 > G5
21	2	2	2	2	2	0.3048
Angiogenesis 3	1	2	2	2	1	0.0169 G3 > G1; G4 > G1 G3 > G5; G4 > G5
7	3	3	3	3	3	1.00
14	2	3	2	2.5	3	0.0297 G5 > G1
21	2	2	2	2	3	0.0224 G5 > G1
Hemorrhage 3	1	1	1	1	0.5	0.0356 G4 > G5
7	1	0	1	1	1	0.3282
14	0	0	0	0	0	0.2692
21	0	0	0	0	0	0.6027

DAI: days after the injury induction;

* values presented are median; G1: neutral control (saline solution); G2: 5% cream-gel; G3: 15% cream-gel; G4: 25% cream-gel; G5: cream-gel. The statistical analysis considered a semi-quantitative analysis as follows: 0: absent; 1: discrete; 2: moderate; 3: accentuated. Statistical test used: Kruskal-Wallis’s test.

**Figure 3:**
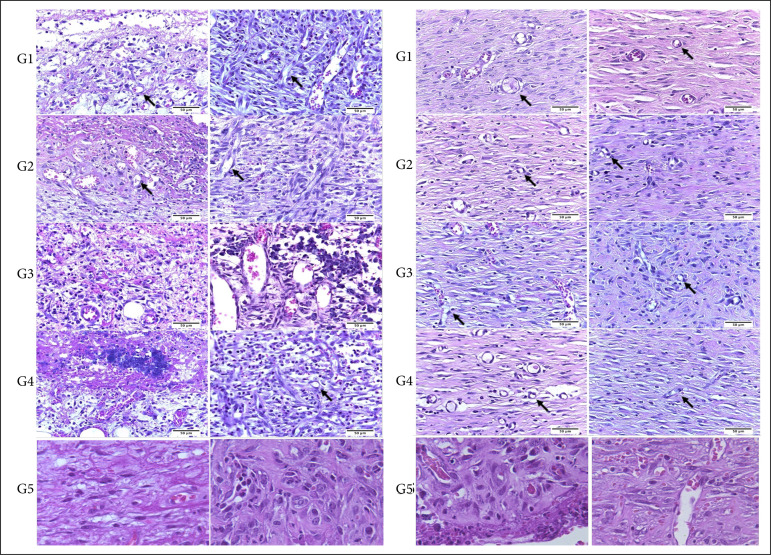
Photomicrograph of the skin of rats which suffered the experimental induction of an excisional wound at three, seven, 14 and 21 DAI. Stain: hematoxylin and eosin. Scale: 50 μm. Arrows indicate the presence of new vessels (angiogenesis).

DAI: days after the injury induction; G1: neutral control (saline solution); G2: 5% cream gel; G3: 15% cream gel; G4: 25% cream gel; G5: cream gel.

Regarding the inflammation rate, it was observed that the number of polymorphonuclear (PN) and mononuclear infiltrates (MN) were significantly higher in G5, and consequently the fibroblast and angiogenesis were significantly lower in G5. Additionally, in relation to the three DAI, it was observed that the number of MN and new blood vessels in G3 and G4 (15 and 25% cream-gel) differed significantly from the control group (p < 0.05). Thus, in the inflammation phase, treatments with cream-gels at 15 and 25% of *H. speciosa* latex induced a significant increase in angiogenesis and in the number of MN in the wounds. Furthermore, in G3 and G4, a greater amount of MN was observed than in G2 (5% latex). In the proliferative phase (seven and 14 DAI) and in the remodeling phase (21 DAI), there was no significant difference between the treated groups and the control group. Figure 3 shows representative histological images of each experimental group.

**Figure 4:**
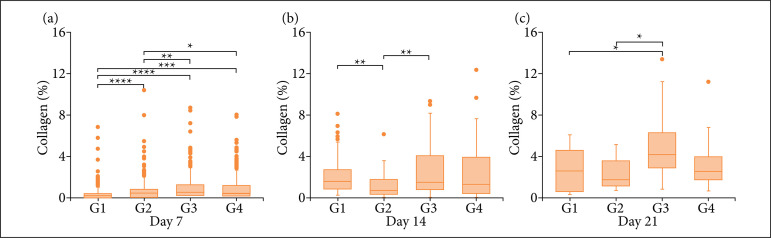
Collagen rate evaluated in excisional wounds of rats treated with saline solution (G1), *Hancornia speciosa* latex-based cream-gel 5% (G2), 15% (G3) and 25% (G4)*. The statistical analysis considered a semi-quantitative analysis as follows: 0: absent; 1: discrete; 2: moderate; 3: accentuated. Statistical test used: Kruskal-Wallis’s test.

DAI: days after the injury induction; *values presented are median; G1: neutral control (saline solution); G2: 5% cream-gel; G3: 15% cream-gel; G4: 25% cream-gel.

Regarding the collagen rate (Fig. 4), our analysis showed that on the seventh DAI there was greater collagen production in all groups treated with cream-gel when compared to control group (G1), and G3 showed the largest collagen production. In the 14th DAI, a decrease in collagen synthesis was observed in G2, when compared to the control. However, the rate of collagen in G2 differed significantly from G3, which demonstrates that individuals treated with 15% cream-gel produced more collagen than individuals who received 5% cream-gel. Finally, in the remodeling phase (21 DAI), G3 synthesized more collagen than group G2, but they did not differ from the control group.

## Discussion

This study addressed the evaluation of healing of excisional wounds experimentally induced in Wistar rats, using as treatment the *H. speciosa* latex-based cream-gel. Previous studies have been performed using the *H. speciosa* latex biomembrane and have shown the natural ability to stimulate the angiogenesis and tissue remodulation^22^^,^ 23 ^,^ 27 ^,^ 28. Despite the promising results using the *H. speciosa* biomembrane, here we chose works with cream-gel because we believe this formulation could facilitate the drug’s application on wounds, as well as avoid the wound bed drying out, which could inhibit the new epithelium formation^33^.

For the cream-gel formulation, we selected the commercial base focus gel 305. During the cream-gel formulation, we removed the rubber fraction from the latex and used the serum aqueous fraction. As the addition of aqueous fraction changed the viscosity of the product, we evaluated different concentrations and selected the concentrations of 5, 10 and 25% m/v, in which the viscosity remained adequate. After cream-gel formulation, we evaluated the healing potential of *H. speciosa* latex-based cream-gel on excisional wounds in rats. To achieve this, we analyzed general macroscopic and microscopic pathological processes after treatment and compared them with a neutral control group.

For the macroscopic analysis, we observed that the treatment with cream-gel (5 and 15%) during the inflammatory phase induced a higher rate of contraction in the wounds. It is known that the rate of contraction depends on the degree of tissue laxity and shape of the wound^34^. Different from humans, who present tight skin, rats possess loose skin, and the contraction process is different, which makes it difficult to compare between both organisms. However, there are some advantages in the use of rats as a research model, such as the availability of a broad knowledge described in the literature^35^.

Studies using wounds in animal models have commonly categorized the wound healing process in the following phases: the inflammatory stage comprising establishment of hemostasis and inflammation; the proliferative stage consisting of granulation, contraction, and epithelization, and the remodeling stage, which exerts influence on the strength and the appearance of the healed tissue^36^.

The results obtained in this research showed that the therapeutic treatment with cream-gel 15 and 25% is beneficial in the inflammatory phase (3 DAI) of the healing process. An increase of the proliferation of MN in the wounds and recruitment of inflammatory cells (Figs. 3 and 4, Table 1) was observed. The inflammatory cells invade the wound site within a few hours after injury. The activation of inflammatory cascade response stimulates the cells secreting several angiogenic factors such as vascular endothelial growth factor A human recombinant (VEGFA), transforming growth factor-ß (TGF-ß), transforming growth factor-1 (TGF-1), platelet-derived growth factor (PDGF), acid aminoacid growth factor (aFGF), among others, which are responsible for stimulating the onset of intense activity angiogenic in tissue^37^.

Thus, in a framework of normal healing, it is expected that in the first 72 hours there is a significant increase in macrophages at the wound site, with maximum vascularization around the fifth day^38^. However, with the progression of the healing process, the initial inflammation stage decreases, and this allows the remodeling phase to advance^38^. This increase in pro-inflammatory cells during an initial period and their orderly control significantly contributes to angiogenesis, the formation of collagen fibers, and, consequently, to the wound healing process^28^. One possible explanation for orderly control inflammation process is the phytochemical composition of *H. speciosa* serum fraction. That latex fraction contains chlorogenic acids, naringenin-7-O-glucoside, catechin, and procyanidin^23^^,^ 24. Chlorogenic acids exhibit antioxidant, antimutagenic, and anti-inflammatory activities and can modulate numerous important metabolic pathways^39^. In addition, it has been reported that naringenin-7-O-glucoside reduces migration and inflammation in human aortic endothelial cells^40^, and that this modulation could be associated with the reduction of pro-inflammatory cytokine expression (interleukin-1β, interleukin-8, and transforming growth factor-α)^41^.

Regarding the cream-gel without serum latex (G5), an increase in necrosis, fibrin and inflammation rates and a decrease in angiogenesis and fibroblast production were observed. As the risk of wound infection increases according to local conditions, the moist wound environment created by the cream without any active ingredient could favor the proliferation of microorganisms and impair the healing process^42^.

Wound contraction begins almost concurrently with collagen synthesis. The most important feature in the remodeling phase is the organized collagen deposition^34^. Collagen is the most abundant protein in the human body and it is the main component of the extracellular matrix of tissues. During the healing process, the collagen produced initially is thinner than the collagen in normal skin and has an orientation parallel to the skin^43^. Over time, the initial collagen is reabsorbed, and thicker collagen is produced and organized along lines of tension^44^. These changes in collagen fibers will increase the tensile strength of the wound and help the wound to contract. This pattern was observed in the present study, as the 15% cream-gel stimulated increased collagen synthesis in the wound bed, generating a higher rate of wound contraction. Besides the increase in collagen synthesis, the remodeling phase also presents reduction in necrosis, inflammatory infiltrates, angiogenesis, migration of fibroblast and hemorrhage (Table 1). Our data agree with that obtained using the *H. speciosa* biomembrane^28^. According Pegorin^28^, the *H. speciosa* biomembrane stimulated the proliferation of inflammatory cells and the formation of new vessels in injured tissues, during the initial phases of the healing process, and they significantly increased collagen synthesis from the 14th day of treatment.

## Conclusions

*Hancornia speciosa* latex-based cream-gel prevents the drying out of wounds and stimulates the contraction of wounds, inflammatory infiltration, and angiogenesis during the initial phase of the wound healing process. It also stimulates collagen production and reduction of inflammatory infiltrates, angiogenesis, migration of fibroblast, and hemorrhage in the proliferative phase. The combination of these properties makes the latex of *H. speciosa* a potential subject of study for the development of low-cost herbal medicines which aid in tissue repair.
